# Medically Treated Opioid Overdoses Among the U.S. Elderly: Trends and Correlates

**DOI:** 10.1093/geroni/igaa057.176

**Published:** 2020-12-16

**Authors:** Peter Treitler, Stephen Crystal, Richard Hermida, Jennifer Miles

**Affiliations:** Rutgers University, New Brunswick, New Jersey, United States

## Abstract

High rates of opioid prescribing and comorbid medical conditions increase risk of overdose among older adults. As the US population ages and the rates of opioid use disorder (OUD) increase in the elderly population, there is a need to characterize trends and correlates of overdose in order to more effectively target policy and practice. Using a ~40% random sample of 2015-2017 Medicare beneficiaries ages 65 and older with Part D pharmacy coverage, this study examined medically treated opioid overdoses among US older adults. The sample included 13-14 million beneficiaries per year. The rate of medically treated opioid overdoses among elderly Medicare beneficiaries increased by 15% from 6 per 10,000 in 2015 to 6.9 per 10,000 in 2017. Those with overdose were disproportionately female (63%), non-Hispanic white (83%), with diagnoses of pain conditions (96%), with diagnoses of major depression (63%), and with high rates of conditions that decrease respiratory reserve such as chronic obstructive pulmonary disease. 13% had co-occurring diagnosed alcohol use disorder, 36% were diagnosed with opioid dependence or abuse, and 12% were diagnosed with hepatitis C. Older individuals with overdose represent a complex mix of risk factors; identifying those most at risk (as well as those who have very low risk, whose pain management may be compromised by overly-rigid interpretation of opioid use guidelines) is key in order to address multiple risks, balancing risk reduction with appropriate pain management.

